# Total Hip Arthroplasty for Acetabular Fracture Sequelae: Surgical Complexity and Technical Pitfalls in a Series of 16 Cases

**DOI:** 10.7759/cureus.103420

**Published:** 2026-02-11

**Authors:** Ilyesse Haichour, Amine El Farhaoui, Sohayb Darraz, Hamza Margoum, Brahim Zeryouh, Achraf Tebbaa El Hassali, Abdeljaouad Najib, Hicham Yacoubi

**Affiliations:** 1 Department of Orthopaedics, Mohammed VI University Hospital Center, Oujda, MAR; 2 Faculty of Medicine and Pharmacy, Mohammed First University, Oujda, MAR

**Keywords:** acetabular fractures, complications of acetabular fractures, hip avascular necrosis, secondary hip osteoarthritis, total hip arthroplasty (tha)

## Abstract

Total hip arthroplasty (THA) performed for sequelae of acetabular fractures remains one of the most technically demanding reconstructive procedures in hip surgery due to post-traumatic anatomical distortion, acetabular bone loss, heterotopic ossification, fibrosis, and altered biomechanical landmarks. These factors are associated with increased intraoperative complexity and higher complication rates compared with primary THA. The purpose of this study was to analyze the surgical challenges, technical pitfalls, complications, and mid-term functional outcomes of THA performed for post-traumatic acetabular sequelae. We conducted a retrospective study including 16 patients who underwent THA between January 2020 and January 2026 at Mohammed VI University Hospital, Oujda, Morocco. The cohort showed a marked male predominance in 14 cases (87.5%), with a mean age of 54.6 years (range, 31-79). According to the Judet and Letournel classification, posterior wall fractures in six cases (37.5%) and transverse fractures in three cases (18.75%) were the most frequent patterns, and in 10 cases (62.5%), patients had an associated hip dislocation. Initial fracture management was surgical in seven cases (43.75%) and conservative in nine cases (56.25%). The mean delay between acetabular fracture and development of degenerative sequelae was 97.5 months. Radiographic evaluation demonstrated post-traumatic osteoarthritis in 13 cases (81.25%), femoral head avascular necrosis in seven cases (43.75%), and acetabular non-union in six cases (37.5%). THA was performed using predominantly double-mobility implants with cemented, cementless, hybrid, or reverse hybrid fixation depending on bone quality and acetabular defects. Intraoperative technical difficulties were encountered in 12 cases (75%), mainly related to loss of anatomical landmarks, acetabular bone loss requiring bone grafting, heterotopic ossification, and fibrosis-related neurological vulnerability. Previous osteosynthesis hardware did not interfere with implant positioning and was preserved in all cases to limit operative time and infectious risk. Postoperative complications included periprosthetic fractures in two cases (12.5%), two reported cases of infections (12.5%), neurological complications in three cases (18.75%), and one prosthetic dislocation (6.25%). At a mean follow-up of 45 months, the Harris Hip Score improved significantly from a preoperative mean of 52.7 to 84.4 at final evaluation. Despite a high rate of surgical complexity and complications, THA for acetabular fracture sequelae provided satisfactory mid-term functional outcomes when meticulous preoperative planning and adapted surgical strategies were applied.

## Introduction

Acetabular fractures are severe injuries that frequently affect young and middle-aged adults following high-energy trauma, often associated with hip dislocation and chondral damage [1]. Despite improvements in fracture fixation techniques and perioperative care, long-term outcomes remain unpredictable, and a substantial proportion of patients develop post-traumatic sequelae requiring secondary total hip arthroplasty (THA) [2].

Post-traumatic osteoarthritis represents the most frequent indication for secondary THA, resulting from residual incongruity, cartilage damage, femoral head ischemia, and imperfect reduction [3]. Avascular necrosis of the femoral head and acetabular non-union further compromise joint integrity and accelerate degenerative changes [4].

Compared with primary THA, THA performed after acetabular fractures is associated with significantly higher complication rates, including infection, dislocation, periprosthetic fracture, and aseptic loosening [5].

Surgical complexity is largely related to altered pelvic anatomy, distorted acetabular orientation, bone defects, heterotopic ossification, and dense fibrotic tissues obscuring standard anatomical landmarks [6]. Neurological complications, particularly involving the sciatic nerve, are more frequent due to scar tissue and altered nerve trajectories following trauma or previous surgery [7]. Implant selection remains controversial, especially regarding cemented versus cementless fixation and the role of double-mobility components in reducing instability [8].

While survivorship and functional outcomes have been reported in several series, fewer studies have specifically focused on operative challenges and technical pitfalls, particularly in developing countries [9].

The purpose of this study was therefore to analyze in detail the surgical complexity, intraoperative difficulties, complications, and mid-term outcomes of THA performed for acetabular fracture sequelae in a tertiary referral center.

## Materials and methods

We conducted a retrospective observational study including patients who underwent THA for sequelae of acetabular fractures between January 2020 and January 2026 at the Department of Traumatology and Orthopaedic Surgery B, Mohammed VI University Hospital, Oujda, Morocco. Patients were eligible if they presented with disabling hip pain and functional limitation associated with radiographic evidence of post-traumatic osteoarthritis, femoral head avascular necrosis, and/or acetabular non-union following an acetabular fracture. Patients undergoing THA for primary osteoarthritis or inflammatory joint disease were excluded. Acetabular fracture patterns were classified according to the Judet and Letournel classification based on initial imaging [10]. Preoperative assessment included a thorough clinical examination and standard radiographic evaluation with anteroposterior pelvic and lateral hip radiographs.

A systematic preoperative infectious work-up was performed in all patients, including complete blood count and C-reactive protein measurement, which showed no biological signs suggestive of infection. In patients with a history of previous surgical treatment of the acetabulum, intraoperative periacetabular tissue samples were systematically collected for microbiological analysis, and all cultures returned sterile, confirming the absence of occult infection. All procedures were performed by senior surgeons from the same orthopedic team using a posterolateral approach. The choice of implant configuration (cemented, cementless, hybrid, or reverse hybrid THA, predominantly using double-mobility cups) was adapted intraoperatively according to bone quality, acetabular defects, and primary implant stability.

Previous osteosynthesis hardware was preserved whenever it did not interfere with acetabular reaming or implant positioning, in order to limit operative time and reduce hemorrhagic and infectious risks. Minor acetabular bone defects were managed using autologous cancellous bone graft harvested from the femoral head, while an allograft was required in one case. No acetabular reinforcement rings were routinely used. All patients received intravenous antibiotic prophylaxis with cefalotin for 48 hours postoperatively, and no surgical drains were used. Postoperative rehabilitation included early mobilization, with full weight-bearing ambulation using a walker for the first 15 days when postoperative radiographs showed no contraindications. Thereafter, patients were encouraged to progressively resume normal daily activities according to pain tolerance and radiological findings.

Clinical outcomes were assessed using the Harris Hip Score on a 100-point scale and based on four domains: pain (44 points), function (47 points) - including gait and activities of daily living - absence of deformity (four points), and range of motion (five points). Higher scores indicate better hip function, with results commonly interpreted as excellent (90-100), good (80-89), fair (70-79), and poor (<70) [11]. The score was recorded preoperatively and at final follow-up. Radiographic evaluation was performed immediately after surgery and at regular intervals during follow-up to assess implant positioning, stability, and the presence of radiolucent lines, migration, or heterotopic ossification. All complications were recorded during the follow-up period, and implant failure was defined as any revision surgery performed for either septic or aseptic causes.

## Results

**Figure 1:**
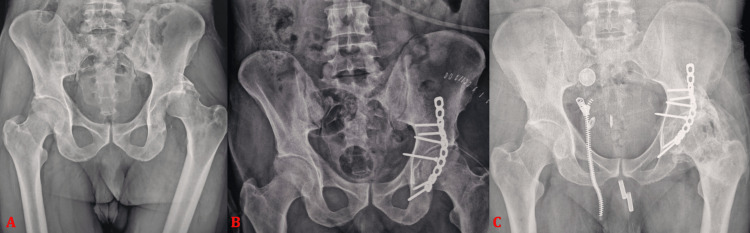
Anteroposterior pelvic radiographs showing (A) a posterior wall fracture of the left acetabulum associated with dislocation of the femoral head in a 38-year-old male patient. The patient underwent open reduction and internal fixation using a plate-and-screw construct, with postoperative control radiograph (B) demonstrating a congruent hip joint space. Follow-up at 23 months revealed progression to post-traumatic hip osteoarthritis with the development of heterotopic ossification (C).

**Figure 2:**
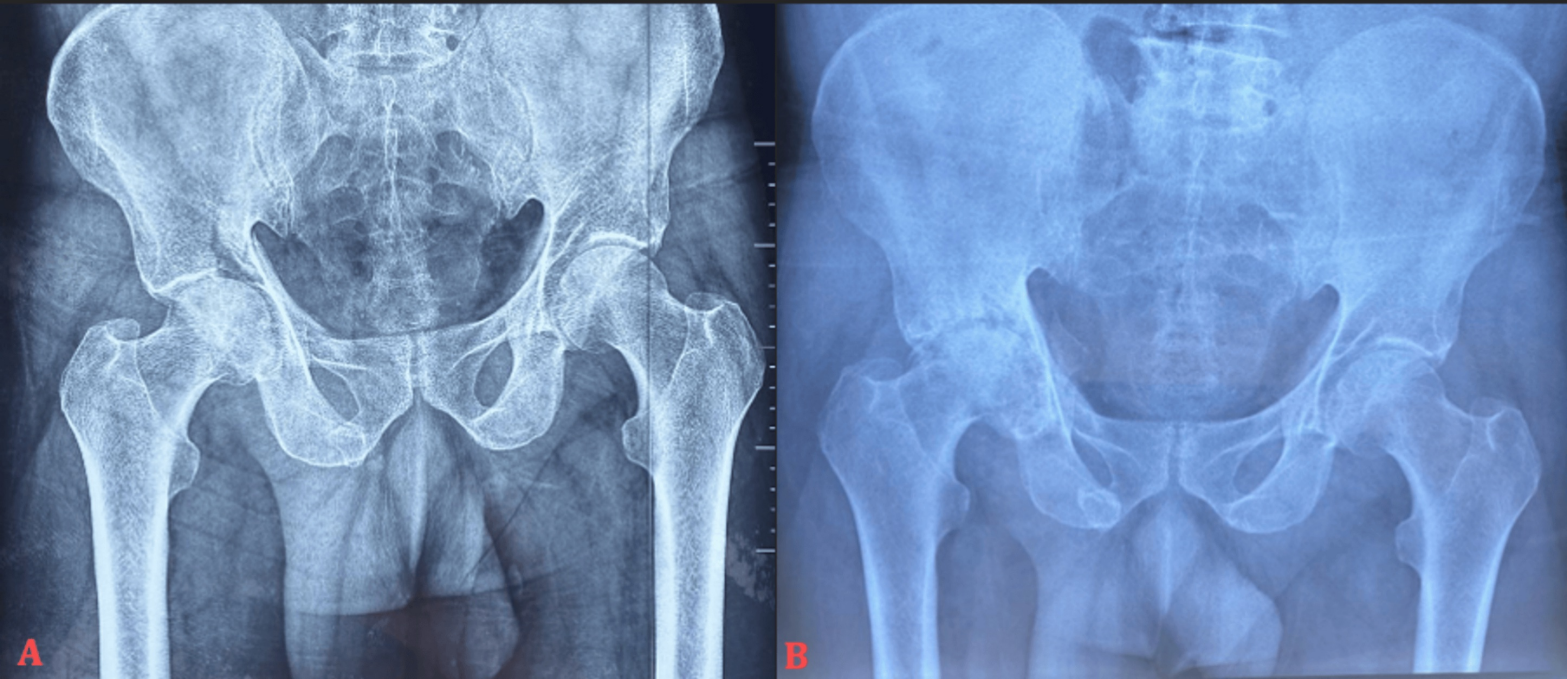
Anteroposterior pelvic radiographs showing (A) an initial transverse acetabular fracture in a 69-year-old male patient and (B) progression at six months to post-traumatic hip osteoarthritis associated with avascular necrosis of the femoral head.

**Figure 3:**
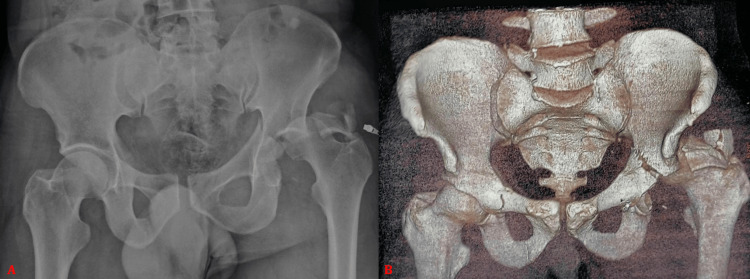
Anteroposterior pelvic radiograph and (B) three-dimensional CT bone reconstruction showing a transverse acetabular fracture with posterior wall involvement according to the Judet and Letournel classification, associated with a posterosuperior dislocation of the femoral head in a 46-year-old male patient.

In our series, the indication for THA was related to post-traumatic sequelae of acetabular fractures in all patients. The study included 16 patients with a marked male predominance 14 cases (87.5%) and a mean age of 54.6 years (range, 31-79 years). According to the Judet and Letournel classification, posterior wall fractures were the most frequent pattern (Figure 1), accounting for six cases (37.5%), followed by transverse fractures in three cases (18.75%) of the patients (Figure 2). An associated hip dislocation was documented in 10 cases (62.5%) at the time of the initial injury (Figure 3). Initial fracture management had been surgical in seven cases (43.75)% while nine cases (56.25%) had been treated conservatively. The mean delay between the initial acetabular fracture and the onset of degenerative or structural sequelae leading to THA was 97.5 months, with a wide range from four to 480 months.

**Figure 4:**
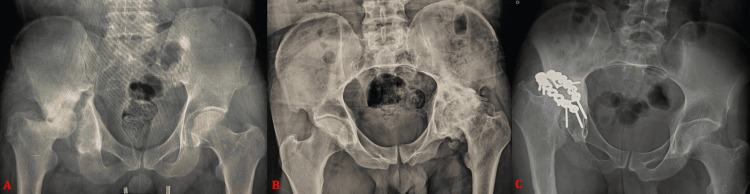
(A) Anteroposterior pelvic radiograph showing sequelae of a transverse fracture of the right acetabulum treated conservatively, with progression to fracture non-union and avascular necrosis of the femoral head in a 31-year-old male patient. (B) Anteroposterior pelvic radiograph demonstrating left post-traumatic hip osteoarthritis associated with heterotopic ossification following a posterior wall fracture-dislocation treated conservatively in a 58-year-old male patient. (C) Anteroposterior pelvic radiograph showing a right acetabular fracture treated by posterior open reduction and internal fixation using two plate-and-screw constructs, with progression at 46 months to post-traumatic hip osteoarthritis associated with femoral head avascular necrosis in a 47-year-old male patient.

Radiographic assessment prior to arthroplasty demonstrated post-traumatic hip osteoarthritis in 13 cases (81.25%), avascular necrosis of the femoral head in seven cases (43.75%), and acetabular non-union in six cases (37.5%) (Figure 4). Preoperative functional evaluation revealed severe functional impairment, with disabling pain, limping, and stiffness in the majority of patients, and a mean preoperative Harris Hip Score of 52.7%. Preoperative laboratory tests, including complete blood count and C-reactive protein, showed no evidence of infection, and systematic intraoperative microbiological samples obtained from previously surgically treated acetabula were all sterile.

**Figure 5:**
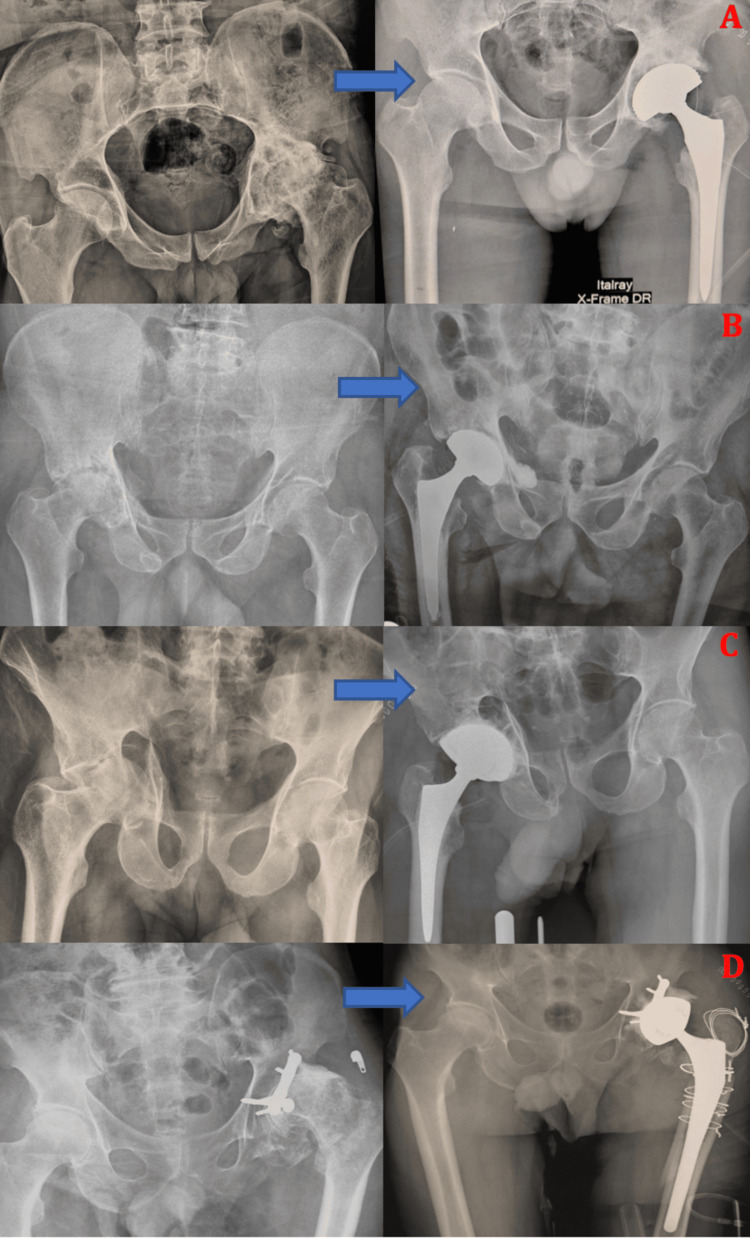
Preoperative and postoperative radiographs of patients from the series after implantation of double-mobility total hip arthroplasties for sequelae of acetabular fractures, illustrating different fixation modes: (A) cementless fixation in a 58-year-old male patient, (B) fully cemented fixation in a 69-year-old male patient, (C) reverse hybrid fixation in a 59-year-old male patient, and (D) hybrid fixation in a 58-year-old male patient.

Surgical management consisted predominantly of double-mobility THA, with cemented fixation in five cases (31.25%), cementless fixation in seven cases (43.75%), hybrid fixation in two cases (12.5%), and reverse hybrid fixation in one case (6.25%) (Figure 5). A single case (6.25%) was treated with a non-cemented ceramic-on-ceramic single-mobility prosthesis.

**Figure 6:**
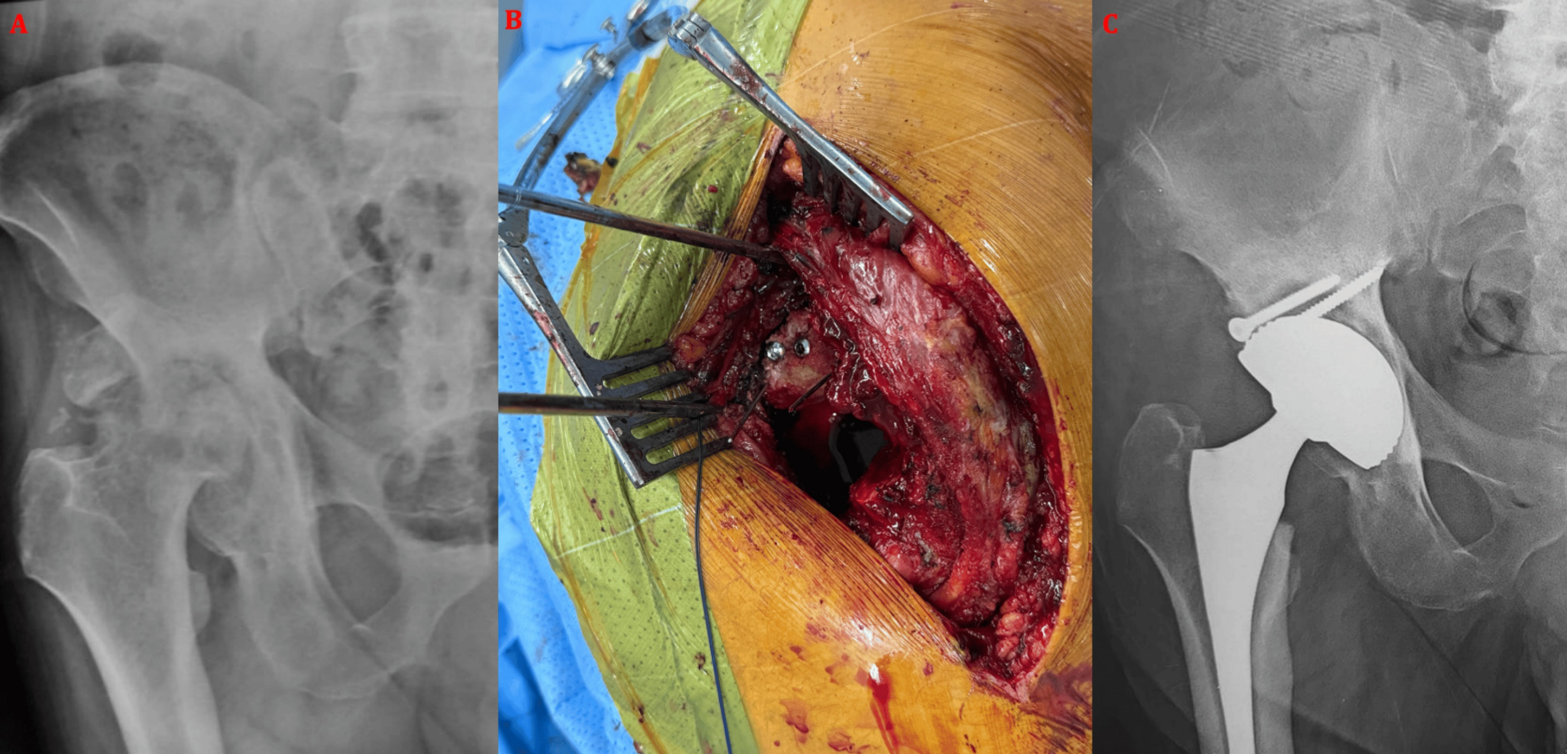
Anteroposterior radiograph of the right hip showing avascular necrosis of the femoral head associated with bone defects of the acetabular roof and posterior wall, as sequelae of a right acetabular fracture initially treated conservatively in a 49-year-old male patient (A). A structural bone allograft was used to fill the acetabular defect and fixed with two screws (B). The postoperative control radiograph demonstrates implantation of the total hip arthroplasty after graft fixation (C).

**Figure 7:**
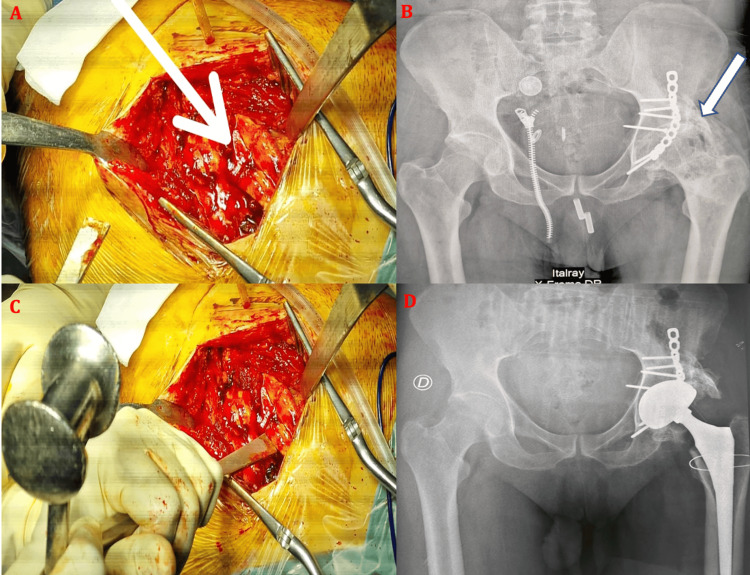
(A) Intraoperative view through a posterolateral Moore approach showing the presence of a bony bridge extending from the greater trochanter to the acetabulum (white arrow), corresponding to heterotopic ossification as demonstrated on the anteroposterior pelvic radiograph (B), in a 38-year-old male patient. Careful release was performed using an osteotome (C) to allow adequate exposure of the pathological hip and subsequent implantation of the total hip arthroplasty (D).

**Figure 8:**
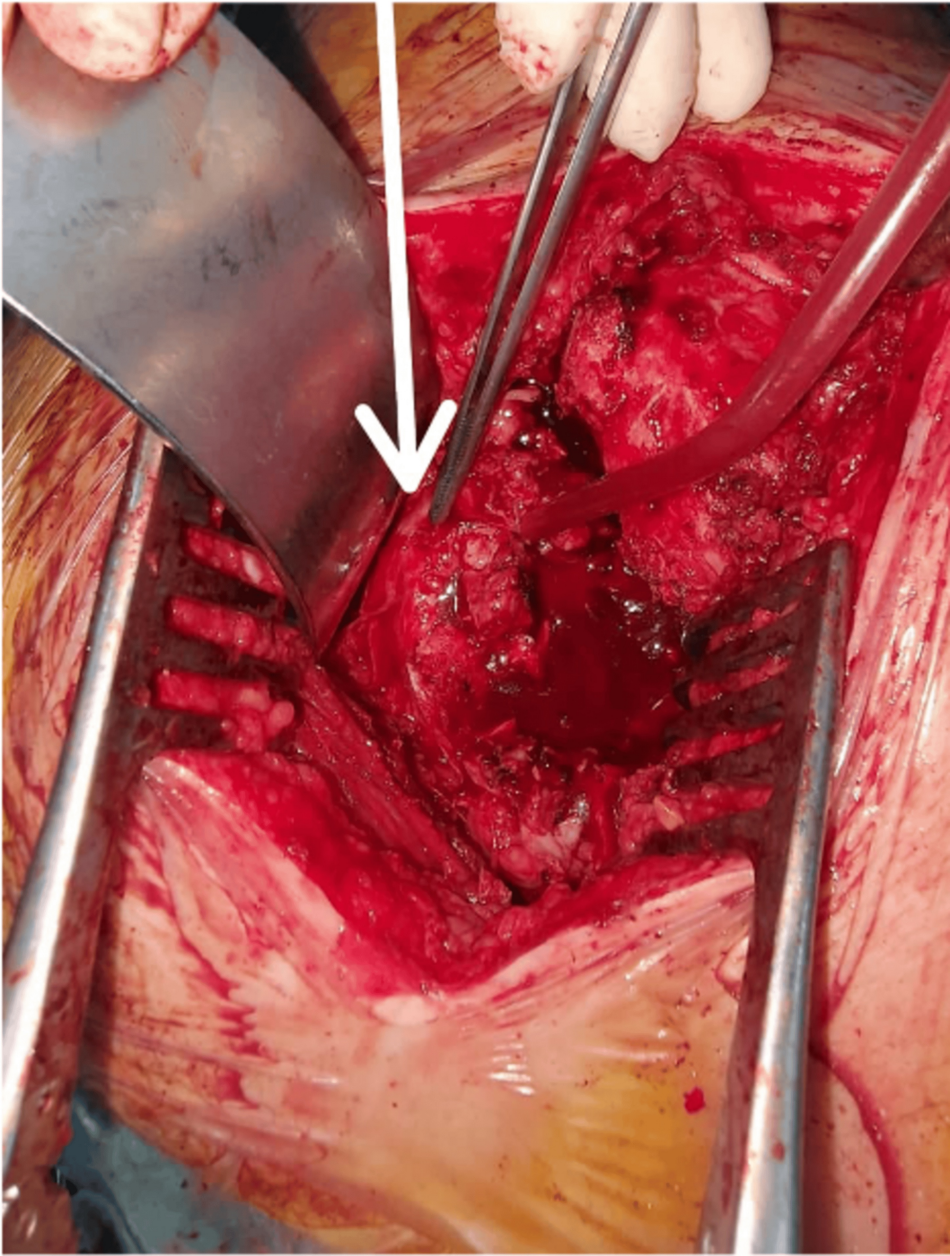
Intraoperative image during total hip arthroplasty performed through a posterolateral approach in a 38-year-old male patient, showing an atypical course of the sciatic nerve (white arrow) passing through dense fibrotic scar tissue and adjacent heterotopic ossifications, rendering it particularly vulnerable to iatrogenic injury due to loss of the usual anatomical landmarks.

Intraoperative technical difficulties were encountered in 12 cases (75%). Minor acetabular bone loss was observed in seven cases (43.75%) and was managed using autologous cancellous bone graft harvested from the femoral head, while one patient required an allograft (Figure 6) without the need for acetabular reinforcement rings. Heterotopic ossifications complicated surgical exposure and implant positioning in three cases (18.75%) (Figure 7). Neurological vulnerability related to post-traumatic fibrosis and altered anatomy was observed intraoperatively in three cases (18.75%) (Figure 8).

The most frequently reported challenge was the loss of standard anatomical landmarks for acetabular orientation and implant positioning, which was noted by the operating surgeons in 75% of cases (12 cases). Previous osteosynthesis hardware did not interfere with acetabular preparation or implant positioning and was therefore preserved in all patients to avoid prolonging operative time and increasing hemorrhagic or infectious risks.

**Figure 9:**
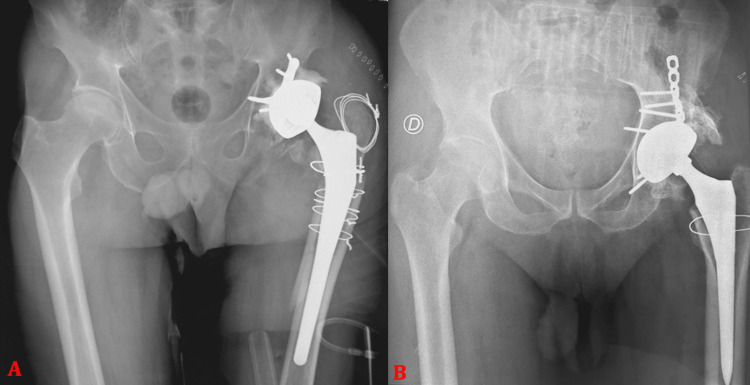
Radiographs of two patients from the series (A and B) showing intraoperative periprosthetic fractures managed with cerclage using stainless steel wires. Image (A) corresponds to a 58-year-old male patient, whereas image (B) illustrates the case of a 38-year-old male patient.

**Figure 10:**
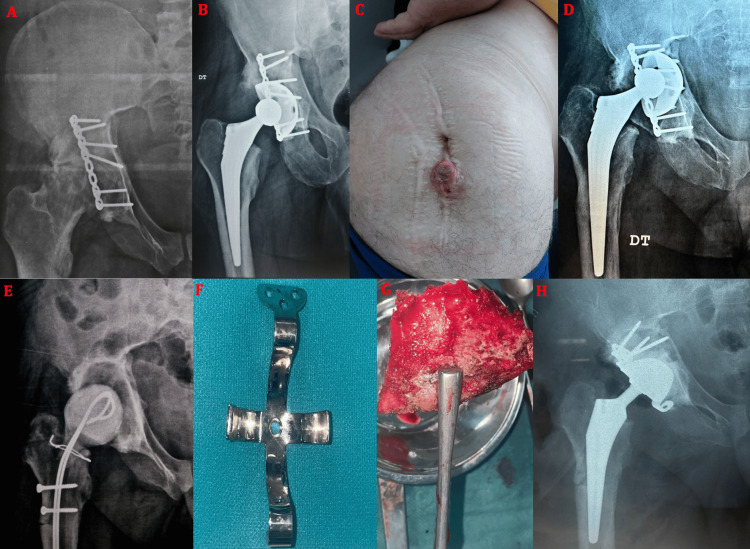
Case of a 46-year-old male patient from the series. (A) Anteroposterior radiograph of the right hip showing post-traumatic hip osteoarthritis associated with avascular necrosis of the femoral head following an acetabular fracture initially treated with plate-and-screw fixation. (B) The patient subsequently underwent cementless double-mobility total hip arthroplasty. Clinical evolution was marked by the development of a draining sinus with purulent discharge 14 months after THA implantation (C), associated with aseptic loosening of the acetabular cup (D). The patient was initially treated with surgical debridement, removal of the prosthesis, placement of an antibiotic-loaded spacer, and targeted antibiotic therapy, followed by secondary reconstruction using a Kerboul acetabular reinforcement ring (F) combined with corticocancellous autograft (G), as demonstrated on the final follow-up radiograph (H).

**Figure 11:**
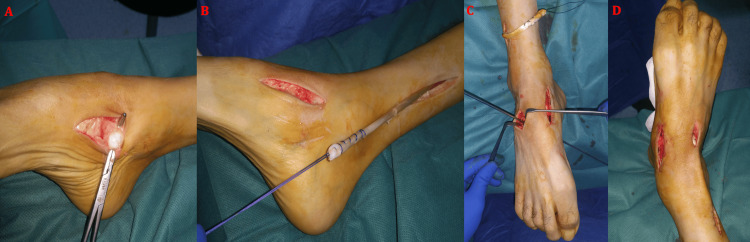
Intraoperative images illustrating the palliative management of sciatic nerve palsy responsible for an equinus deformity incompatible with ambulation in a 59-year-old male patient, treated by posterior tibial tendon transfer. (A) Identification and distal harvesting of the posterior tibial tendon from its insertion site. (B) Proximal release of the tendon followed by preparation of its new fixation site on the dorsal aspect of the third cuneiform (C). (D) Correction of the equinus deformity and final fixation of the transferred tendon.

Postoperative complications were recorded in 37.5% of the patients. Periprosthetic fractures occurred in 12.5% of cases and were successfully managed intraoperatively with cerclage wiring (Figure 9). Two patients (12.5%) developed postoperative infections; one was treated with surgical debridement, targeted antibiotic therapy, and implant retention, while the other required a more complex management including prosthesis removal, spacer placement, prolonged antibiotic therapy, and subsequent revision using an acetabular reinforcement ring due to significant bone loss (Figure 10). Neurological complications were observed in three patients (18.75%), including two cases of neurapraxia that resolved favorably and one case of complete sciatic nerve palsy that required palliative management with posterior tibial tendon transfer (Figure 11). One prosthetic dislocation (6.25%) occurred and was successfully treated with closed reduction under general anesthesia, with no recurrence during follow-up.

At a mean follow-up of 45 months, functional outcomes showed a significant improvement, with the mean Harris Hip Score increasing from 52.7% preoperatively to 84.4% at the last evaluation. Most patients reported substantial pain relief and improved walking capacity. Radiographic follow-up demonstrated stable implant fixation in the majority of cases, with no progressive radiolucent lines or signs of mechanical loosening, except in the case requiring revision surgery. Overall, despite the high rate of technical complexity and perioperative challenges, THA for acetabular fracture sequelae provided satisfactory mid-term functional outcomes in this series.

Table 1 summarizes the demographic characteristics and preoperative clinical and radiographic findings of the patients included in this study. Intraoperative technical challenges, short- and mid-term postoperative complications, and clinical outcomes are summarized in Table 2.

**Table 1:** Demographic characteristics and preoperative clinical and radiographic findings of the study population

	Value
Sex	
Male	14 (87.50%)
Female	2 (12.50%)
Age (years)	54.6 ± 15.4
Initial fracture pattern	
Posterior Wall	6 (37.50%)
Posterior Column	2 (12.50%)
Posterior Column + Posterior Wall	2 (12.50%)
Transverse	3 (18.75%)
Transverse + Posterior Wall	1 (6.25%)
Anterior Column	1 (6.25%)
"T" type fracture	1 (6.25%)
Initial hip dislocation	
Presence of dislocation	10 (62.50%)
Absence of dislocation	6 (37.50%)
Treatment for the acetabular fracture	
Surgery	7 (43.75%)
Conservative	9 (56.25%)
Delay between the treatment and complications (months)	97.5 ± 129.7
Clinical complications	
Pain	15 (93.75%)
Stiffness	9 (56.25%)
Limp	14 (87.50%)
Radiological findings	
Hip osteoarthritis	13 (81.25%)
Avascular necrosis	7 (43.75%)
Non union	6 (37.50%)
Laboratory findings	
Signs in favor of infection	0 (0%)
No sign in favor of infection	16 (100%)
Preoperative Harris Hip Score	52.7 ± 15.5

**Table 2:** Intraoperative technical challenges, postoperative complications, and clinical outcomes findings of the study population.

	Value
Type of THA	
Dual mobility	15 (93.75%)
Single mobility (Ceramic-on-ceramic)	1 (6.25%)
Fixation mode	
All cemented	5 (31.25%)
Cementless	8 (50%)
Hybrid fixation	2 (12.5%)
Inverted hybrid fixation	1 (6.25%)
Microbiological per-operative samples	
Sterile	16 (100%)
Infection	0 (0%)
Hardware of the previous osteosynthesis	
Kept	7 (43.75%)
Removed	0 (0%)
Bone loss	
Minor treated with autologous cancellous bone graft	7 (43.75%)
Major treated with structured allograft	1 (6.25%)
Acetabular reinforcement rings	0 (0%)
Heterotopic ossifications	3 (18.75%)
Postoperative neurological complications	
Neurapraxia	2 (12.50%)
Sciatic nerve palsy	1 (6.25%)
Postoperative infection	2 (12.5%)
Total hip arthroplasty dislocation	1 (6.25%)
Postoperative Harris Hip Score	84.40 ± 6.82

## Discussion

Post-traumatic sequelae following acetabular fractures remain a major challenge despite advances in fracture management. According to the Judet and Letournel classification, certain fracture patterns are consistently associated with a higher risk of post-traumatic osteoarthritis and secondary THA. Posterior wall fractures, transverse fractures, and combined fracture patterns involving both columns have been identified as the most arthrogenic lesions, particularly when joint congruency cannot be perfectly restored [12,13]. These fracture types are frequently associated with cartilage damage, marginal impaction, and residual instability, which predispose to early degenerative changes. In our series, posterior wall fractures and transverse fractures predominated, which is consistent with the literature and may partly explain the high rate of post-traumatic osteoarthritis observed.

The presence of an associated hip dislocation plays a critical role in the development of post-traumatic complications. Several studies have demonstrated that fracture-dislocations significantly increase the risk of chondral injury, femoral head ischemia, and avascular necrosis, particularly when reduction is delayed [14,15]. In addition, hip dislocation reflects the severity of the initial trauma and is often associated with complex fracture patterns and extensive soft-tissue damage. In our cohort, more than half of the patients presented with an associated dislocation, which likely contributed to the high incidence of osteoarthritis and femoral head necrosis observed during follow-up.

The delay between fracture treatment and the onset of symptoms such as pain, stiffness, and functional limitation is highly variable. Previous series have reported symptom onset ranging from a few months to several years after the initial injury, depending on fracture pattern, quality of reduction, and initial management [16]. In our study, the mean delay was prolonged, exceeding eight years in some cases, highlighting the progressive and insidious nature of post-traumatic degeneration following acetabular fractures. This delayed presentation underscores the importance of long-term follow-up in this patient population.

The role of THA in the acute management of complex acetabular fractures has been increasingly discussed in recent years. Acute THA, either alone or combined with limited internal fixation, has been proposed in selected elderly patients with comminuted fractures, severe cartilage damage, or poor bone quality, with the aim of allowing early mobilization and avoiding secondary procedures [17,18]. However, this strategy remains technically demanding and is associated with its own complications. In younger and middle-aged patients, delayed THA following fracture healing or failed osteosynthesis remains the preferred approach, as it allows better assessment of bone stock, fracture consolidation, and infection status.

Delayed THA after initial osteosynthesis offers several advantages. It allows restoration of acetabular anatomy, facilitates biological healing, and provides a more stable foundation for subsequent arthroplasty [19]. In addition, performing THA in a healed acetabulum reduces the risk of early loosening and facilitates implant positioning. In our series, patients who had undergone previous surgical fixation appeared to have better bone stock and more predictable reconstruction than those treated conservatively, in whom higher rates of non-union and bone defects were observed.

Our findings support the growing body of evidence advocating for surgical management of displaced acetabular fractures whenever feasible. Several authors have reported higher rates of post-traumatic osteoarthritis and secondary THA in patients treated non-operatively, particularly in fractures involving the posterior wall or transverse patterns [20,21]. In our cohort, a substantial proportion of patients initially treated orthopedically developed severe degenerative changes, reinforcing the importance of appropriate fracture fixation in reducing long-term complications.

Implant selection remains a key issue in THA after acetabular fractures. Double-mobility cups have gained popularity in this setting due to their ability to reduce instability, which is one of the most frequent complications reported in post-traumatic THA [22]. Both cemented and cementless fixation have been described, with cementless cups generally preferred in younger patients with adequate bone stock, while cemented fixation may be advantageous in cases of compromised bone quality [23]. In our series, double-mobility implants were predominantly used, contributing to a low rate of postoperative dislocation.

Management of acetabular bone loss represents another major challenge. Minor defects can be addressed with morselized autograft, while larger defects may require structural grafts or reinforcement devices [24]. Acetabular reinforcement rings, such as Kerboul or Burch-Schneider rings, are indicated in cases of major segmental bone loss, pelvic discontinuity, or insufficient host bone for primary cup stability [25]. In our experience, reinforcement rings were reserved for complex reconstructions and revision settings.

The choice of surgical approach is also crucial. The posterolateral approach is commonly used and allows extensile exposure, particularly in the presence of posterior wall involvement or previous posterior fixation [26]. However, it is associated with an increased risk of instability and sciatic nerve injury, especially in the presence of fibrosis and heterotopic ossification. Careful dissection and systematic identification of the sciatic nerve are therefore mandatory.

Routine removal of previous osteosynthesis hardware is not systematically recommended. Several authors have shown that unnecessary hardware removal increases operative time, blood loss, and infection risk without clear benefit, unless the implants interfere with cup placement or infection is suspected [27]. In our series, hardware was preserved whenever possible, which helped limit surgical morbidity.

Reported complications following THA after acetabular fractures include infection, dislocation, periprosthetic fracture, nerve injury, and aseptic loosening, with rates consistently higher than those observed after primary THA [5,28]. Loss of anatomical landmarks represents a major technical pitfall and requires reliance on preoperative imaging, intraoperative fluoroscopy, and careful assessment of pelvic orientation. Heterotopic ossification is another frequent issue and may complicate exposure; prophylaxis with radiation therapy or non-steroidal anti-inflammatory drugs should be considered in high-risk patients [29].

Immediate postoperative complication rates reported in the literature range from 15% to 30%, depending on patient selection and surgical technique [28]. Our study allowed a detailed assessment of intraoperative and early postoperative complications, as well as mid-term outcomes, highlighting the complexity of these procedures. However, the main limitation of our work remains the absence of long-term follow-up, which is necessary to fully evaluate implant survivorship and late complications.

## Conclusions

THA for sequelae of acetabular fractures is a technically demanding procedure due to post-traumatic anatomical distortion, bone loss, heterotopic ossification, and loss of reliable surgical landmarks. In our series, these factors resulted in a high rate of intraoperative difficulties and complications; however, THA provided significant pain relief and meaningful functional improvement at mid-term follow-up, as reflected by the substantial increase in Harris Hip Score.

These findings highlight the importance of meticulous preoperative planning, experienced surgical teams, and individualized implant selection when addressing post-traumatic acetabular pathology. The use of adapted surgical strategies, including double-mobility components and conservative management of retained hardware, allowed satisfactory clinical outcomes despite the complexity of these cases. Further studies with larger cohorts are needed to better define optimal surgical approaches and long-term outcomes in this challenging patient population.
